# Use of mucolytics and inhaled antibiotics in the NICU

**DOI:** 10.1038/s41372-024-02178-w

**Published:** 2024-11-19

**Authors:** Alexander I. Gipsman, Anita Bhandari, Vineet Bhandari

**Affiliations:** 1 Division of Pulmonary and Sleep Medicine, Children’s Hospital of Philadelphia, Philadelphia, PA USA; 2 Perelman School of Medicine, University of Pennsylvania, Philadelphia, PA USA; 3 Division of Neonatology, The Children’s Regional Hospital at Cooper, Camden, NJ USA; 4 Cooper Medical School of Rowan University, Camden, NJ USA

**Keywords:** Drug delivery, Respiration

## Abstract

Clearance of airway secretions and treatment of respiratory tract infections (RTIs) are two common problems caregivers face in the neonatal intensive care unit (NICU). Mucolytics degrade crosslinks in mucus gel, reducing mucus viscosity and facilitating their removal by cough or endotracheal suctioning. While such medications have been studied in older children and adults, their use is not as well described in the NICU. For RTIs, systemic antibiotics are usually prescribed, although their use is often associated with adverse effects. Inhaled antibiotics may provide increased drug concentrations to the infected airways while minimizing systemic toxicity. The use of inhaled antibiotics in the NICU has been described in small case series. As underlying physiologic differences will lend to inaccuracies when extrapolating data obtained from older children, there is an urgent need to determine the safety, efficacy, and optimal dosing of inhaled mucolytics and antibiotics in infants of varying gestational and post-natal ages.

## Overview of mucus and normal airway clearance mechanisms

Mucus is a viscoelastic gel lining the airway surface comprised of water, mucins, antimicrobial and immunomodulatory molecules, and lipids [1]. It is produced by the secretory cells of the airway epithelium and submucosal glands [1, 2]. The periciliary layer of serous fluid lies beneath the mucus gel layer, and together these form the bilayer airway surface liquid [2, 3]. The primary function of the mucus gel is to trap inhaled material, cellular debris, and microbes and to transport them out of the lungs through ciliary beating and cough [1, 4]. The periciliary layer likely functions to lubricate the cilia and enable them to beat more efficiently [3]. Secretion clearance from the lower respiratory tract occurs by two mechanisms: mucociliary clearance and cough. Mucociliary clearance occurs by a complex coordination of ciliary beat activity, propelling mucus proximally towards the mouth until it is either expectorated or swallowed [3]. The rate of mucociliary clearance is affected by the length and function of the cilia themselves, serous fluid depth, and mucus gel depth and viscoelasticity [5]. When ciliary clearance fails to clear secretions, a cough is utilized to remove secretions from the central airways.

Mucus hypersecretion and changes in the biophysical properties of mucus can both impair and overwhelm ciliary and cough clearance [6], leading to airflow obstruction, atelectasis, hypoxemia due to ventilation-perfusion mismatch, respiratory tract infections (RTIs), and if it persists, permanent airway damage manifesting as bronchiectasis. Infection and inflammation stimulate further mucus secretion which leads to a vicious cycle if not addressed [6]. Intrinsic properties of mucus that may impair its clearance include hydration, addition of molecules associated with inflammation such as DNA and actin, and the degree of polymerization of the mucus gel [6].

## Unique anatomic and physiologic considerations in infants

There are several unique physiologic considerations in infants that may impact innate secretion clearance and inhaled medication delivery. These considerations make it extremely difficult, if not impossible, to extrapolate data regarding the dose, safety, and efficacy of inhaled medications from studies performed in older children and adults.

In children younger than 2 years old, rib orientation is more horizontal, and the diaphragm domes lie higher relative to older children and adults. This limits thoracic expansion and the ability to increase tidal volume in the setting of increased ventilatory demand [7]. Chest wall compliance (C_w_) is significantly higher (i.e., outward chest recoil is low) relative to lung compliance (C_L_) in infants until around two years of life [8]. Because of this, during active (rapid eye movement or REM) sleep when intercostal muscles are relaxed, the chest wall is drawn inwards during inspiration, creating a paradoxical pattern of breathing where the abdomen moves outward and the chest wall inward. Therefore, tidal volume and minute ventilation are reduced during active sleep in young infants [9]. In preterm infants, paradoxical breathing occurs during all stages of sleep, likely due to an even more compliant chest wall [10]. An increased load on the respiratory system easily leads to indrawing of the chest wall during inspiration, which limits the ability to increase tidal volume.

To compensate for increased C_w_, young infants attempt to elevate functional residual capacity (FRC) actively by limiting expiratory time through increasing their respiratory rate, laryngeal braking (laryngeal adduction during expiration), and post inspiratory activity of the diaphragm [11–13]. Dynamic maintenance of end expiratory volume continues until about 6-12 months of life [11, 12]. This seemingly unpredictable activity of the laryngeal muscles and diaphragm are likely have an impact on the delivery of aerosolized medications [12].

Premature infants have a relatively increased amount of bronchial smooth muscle compared to term infants of a similar post-conceptional age, and ventilated infants have an even greater increase in airway smooth muscle. This would exacerbate premature airway closure because of increased expiratory flow resistance [14]. Early airway closure and diminished airway caliber will affect ventilation heterogeneity and have a significant impact on distribution of aerosolized particles [12].

Infants preferentially breathe through their noses [15]. Nasal breathing markedly reduces lung deposition of aerosolized particles because of nasal filtration [16, 17]. Lower tidal volumes and rapid respiratory rates in infants result in a marked reduction of aerosol entering the lungs [18] and reduced pulmonary residence time of particles [19]. Additionally, dead space is relatively large in infants because of their large head size [20]. This, combined with lower tidal volumes, increases the amount of drug deposited in dead space rather than the peripheral airways [12]. Intrinsic anatomic properties of the pharynx in young children and reduced inspiratory flows may be responsible for the finding that aerosol deposition in the upper oropharynx is increased in young children and decreases with increasing age [21].

As most aerosolized particles are delivered to the lungs during inspiration, the ratio of inspiratory to total respiratory cycle time (t_i_/t_tot_), also referred to as duty cycle, is critically important. Decreased duty cycle leads to a reduction in delivery of aerosolized particles to the lung periphery [12, 22]. When obstructive lung disease is present, such as in an infant with severe bronchopulmonary dysplasia (BPD), duty cycle will be reduced dramatically, because the infant must increase expiratory time in order to empty the lungs. This reduces the amount of inhaled drug that reaches the lungs. Additionally, if an infant is crying during drug nebulization, duty cycle is markedly reduced and almost no drug particles will be inspired [12]. In infants with severe BPD, lung disease may be markedly heterogenous with units of varying resistance and compliance (i.e., time constants) [23, 24]. Predicting ventilation distribution, and therefore aerosol particle deposition, is complex and close to impossible. Additionally, based on the patient’s clinical status and recent therapies (e.g., bronchodilator, diuretic medications), ventilation distribution may change several times in a given day [12]. Furthermore, many of these infants have tracheobronchomalacia [25]. A recent imaging study showed that aerosolized drug delivery to the lung parenchyma was reduced in neonates with tracheomalacia [26].

Alveolar pores of Kohn, interbronchiolar Martin’s channels, and bronchoalveolar Lambert’s channels provide pathways for collateral ventilation and preserve gas exchange in the setting of bronchial obstruction. However, these pathways are mostly absent in infants, which predisposes them to respiratory failure, atelectasis, and alteration of aerosol distribution [12, 27, 28]. By 16 weeks’ gestation, all airways are formed, and thereafter increase in size but not in number. Alveoli, in contrast, increase both in size and number after birth. Therefore, airways and alveoli develop in a disproportionate (i.e. dysanaptic) fashion in young children, and this relationship likely impacts aerosol deposition [12].

In infancy and childhood, ventilation is preferentially distributed in upper lung zones, away from dependent lung regions [29, 30]. This pattern is the opposite of what is seen in adults and older children [12, 31]. This would be expected to have a significant impact on aerosol distribution in infants [12].

Finally, the choice of the devices used for aerosolization, specific drug-device combination, presence and type of mechanical ventilation, and ventilator circuit may all have substantial effects on drug deposition in neonates [19].

## Inhaled mucolytic medications

**Fig. 1:**
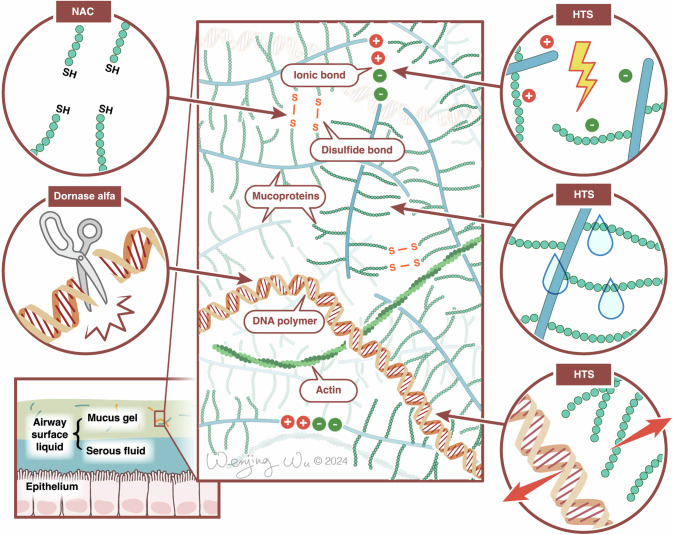
Mucolytics and their mechanisms of action. HTS disrupts ionic bonds between mucoproteins, dissociates DNA from mucoproteins, and hydrates mucus via osmosis. NAC cleaves covalent disulfide bonds between mucoprotein monomers. Dornase alfa cleaves DNA polymers. HTS hypertonic saline, NAC N-acetylcysteine.

Mucolytics are medications that degrade crosslinked molecules within mucus [32]. There are several types of interactions that occur between mucins to form the mucus gel structure, including covalent disulfide bonds, hydrogen bonds, ionic bonds, and intermingling entanglements of macromolecules. When pathologic inflammation is present, a secondary network of leukocyte DNA and actin filaments is formed [5]. Mucolytic medications work by disrupting these crosslinks and thus altering the physical properties of mucus [5]. Fig. 1 summarizes the mechanisms of action of the various mucolytic medications. Optimal mucociliary and cough clearance depend on a particular balance between elasticity and viscosity. Reducing elasticity of mucus would be expected to improve mucociliary clearance but can impair cough clearance [2, 33, 34]. Therefore, if secretions become excessively thin, cough clearance may be impaired. In general, mucolytic medications should only be considered if secretions are excessively thick such that they are thought to impair mucociliary and/or cough clearance, or if they are difficult to clear via suctioning of an endotracheal tube (ETT). Their use is not recommended if secretions are of normal viscosity. As outlined below, the literature reporting the use of inhaled mucolytic medications in the neonatal population is sparse and consists mostly of uncontrolled and/or observational studies, making it difficult to be relied on completely in clinical practice.

### Recombinant human DNase

Recombinant human DNase (dornase alfa) is a mucolytic originally studied in children with cystic fibrosis (CF). It hydrolyzes DNA polymers, which reduces DNA length and therefore sputum viscosity [33, 35]. It is important to refrain from extrapolating data from studies where dornase alfa was used to treat older children to support its use in high-risk neonates. As an example, to support this cautionary approach, while dornase alfa is an effective mucolytic in CF, its use is associated with more exacerbations and increased lung function decline in adults with non-CF bronchiectasis [36].

### Mucus plugging and/or persistent atelectasis

The use of dornase alfa in premature infants was first reported in a case series by Reiter et al. in 2000. Seven extremely low birthweight preterm infants with evolving or established BPD were treated with intratracheal dornase alfa one to two times daily. Authors reported improvement in supplemental oxygen requirement, character of respiratory secretions, and reduction in mean airway pressure. No adverse events occurred [37]. One year later, El Hassan et al. described rapid clinical and radiographic improvement of atelectasis in three mechanically ventilated infants who received intratracheal or nebulized dornase alfa [38]. MacKinnon et al. have reported on the successful use of dornase alfa to treat atelectasis and facilitate extubation in four ventilated premature neonates [39].

In a prospective study, Erdeve et al. administered nebulized dornase alfa to 12 newborns (10 born preterm) with atelectasis that did not improve with standard therapy. Those who did not improve after three days of nebulized dornase alfa received a single endotracheal dose. Ten patients experienced an improvement in chest radiograph scores, respiratory rate, and gas exchange measures with nebulized dornase alfa. The remaining two improved after the dose of endotracheal dornase alfa [40]. Fedakar et al. performed a prospective study in 22 term and preterm infants with either respiratory distress syndrome or transient tachypnea of the newborn admitted to a NICU in Saudi Arabia with persistent atelectasis. Eighteen patients experienced an improvement in chest radiograph scores, respiratory rate, fraction of inspired oxygen (FiO_2_), and pCO_2_ after three days of nebulized dornase alfa. The remaining four patients received endotracheal dornase alfa in addition to nebulized dornase alfa, and they then experienced improvement. There were no adverse events related to dornase alfa therapy [41]. The main limitation of this study was the absence of a control group.

Dornase alfa hydrolyzes DNA polymers that form after leukocyte degeneration, most prominently when neutrophils undergo a mechanism of cell death (NETosis) and release neutrophil extracellular traps [42]. Prodhan et al. therefore sought to understand if dornase alfa was more effective in treating atelectasis when tracheal aspirates (TA) have more polymorphonuclear neutrophils (PMNs) or bacteria present. In this study, researchers retrospectively reviewed records of 38 mechanically ventilated children (median age 3.5 months) with congenital heart disease who received nebulized dornase alfa to treat atelectasis. They found that chest radiograph scores improved significantly after dornase alfa treatment and had a greater effect in those with at least moderate amounts of PMNs and/or bacteria in their pre-treatment TA. No improvement was seen beyond the first 10 doses [43].

Riethmueller et al. examined the efficacy of dornase alfa in *preventing* rather than treating atelectasis. Eighty-eight infants (median age 3.3 months) with congenital heart disease were randomized to receive dornase alfa or placebo instilled into the ETT following cardiac surgery, until extubation. Of note, premature infants were excluded from this study. Subjects who received dornase alfa experienced shorter ventilator times (median 82 vs. 52 h), less atelectasis (17 vs. 6 subjects), and shorter length of ICU stay (median 8 vs. 7 days) [44].

Two studies compared dornase alfa to hypertonic saline (HTS) in the treatment of newborns with atelectasis. Dilmen and colleagues randomized 40 neonates (38 born preterm) with persistent atelectasis to receive either nebulized dornase alfa or 3% HTS. Subjects who received HTS had more atelectasis resolution after three days of treatment and a greater change in oxyhemoglobin saturation (SpO_2_) after each treatment. Neither group experienced any significant adverse events. Notably, there was no control group [45]. Altunhan et al. performed a retrospective study of 87 mechanically ventilated newborns with a variety of underlying conditions who had persistent atelectasis despite usual care, and compared outcomes between subjects who received no inhaled therapy, 7% HTS, dornase alfa, and both 7% HTS and dornase alfa. The group that received both 7% HTS and dornase alfa had the highest proportion of infants who experienced resolution of atelectasis after three days of treatment, lowest median duration of treatment required to achieve resolution of atelectasis, best chest radiograph scores, and the largest pretreatment and post-treatment difference in pCO_2_, respiratory rate, FiO_2_, and peak inspiratory pressures. Authors report several cases of “sudden deterioration” that occurred in all treatment groups but do not further characterize these events. Of note, patients did not receive a bronchodilator prior to administration of 7% HTS, and therefore may have been prone to bronchospasm [46].

#### RTIs

A pilot randomized clinical trial (RCT) comparing seven days of dornase alfa to sham therapy in 11 preterm infants aged zero to four months with ventilator-associated RTI found dornase alfa to be safe and associated with an improvement in supplemental O_2_ requirement, although this did not reach statistical significance. Notable exclusion criteria included extremely ill infants, infants with cyanotic congenital heart disease, and those with congenital malformations of the respiratory system [47]. The small sample size of this study limits making firm conclusions about safety and efficacy in this patient population.

### Hypertonic saline (HTS)

HTS (most commonly 3%) enhances secretion clearance in several ways. It exhibits mucolytic function by two mechanisms. Firstly, it disrupts ionic bonds within the mucus gel, thus reducing crosslinking and viscosity. Furthermore, it dissociates DNA from mucoproteins, allowing natural proteolytic enzymes to degrade the mucoproteins [5, 48, 49]. It also increases airway surface liquid depth via osmosis, thus hydrating mucus and decreasing its viscosity and improving mucociliary clearance. Its irritating effect on the airways triggers cough which clears secretions from central airways [50, 51]. HTS also exhibits antibiofilm properties in vitro [48], which would, in theory, be particularly advantageous in infants with chronic endotracheal or tracheostomy tube dependence [52, 53].

In assessing whether HTS could prevent development of ventilator associated pneumonia (VAP), Ezzeldin and colleagues randomized 100 premature, mechanically ventilated newborn infants (median gestational age 31 weeks) to receive 3% HTS twice daily or standard care. The HTS group experienced significantly less VAP (18%) compared to controls (52%, *p* = 0.001). Duration of mechanical ventilation was also reduced (10.7 vs. 16.9 days, *p* < 0.001). Additionally, subjects who received HTS had less positive TA (22% vs. 64%, *p* < 0.001) and blood (18% vs. 40%, *p* = 0.02) cultures. HTS was well-tolerated and not associated with any significant adverse effects [54]. The lack of blinding in this study is a substantial weakness, particularly because prevention of VAP may be impacted by careful hand hygiene and sterile suctioning practices [55].

Two studies mentioned above suggest that HTS may be useful in treating atelectasis in infants, although they are substantially limited by a lack of control group in one and being of an observational nature in the other [45, 46].

### N-acetylcysteine (NAC)

NAC cleaves disulfide bonds between mucin glycoprotein monomers, reducing mucus viscosity in vitro [56]. However, its use has generally been limited by its propensity to cause bronchospasm and its foul odor (which may cause nausea and vomiting) [57]. Only a few studies examining its use in infants have been reported. In one study, King et al. instilled 0.1–0.2 mL of NAC hourly into ETTs of thirteen infants to maintain tube patency. After removal of the ETT, there was no significant narrowing of the lumen with mucus, and the treatment was well-tolerated [58]. In 1992, Bibi and coworkers randomized 10 mechanically ventilated preterm infants with increased airway secretions to receive directly instilled endotracheal NAC or saline placebo. The use of NAC was associated with a significant increase in total airway resistance by the third day of therapy. There was no significant difference in gas exchange measures, peak inspiratory pressures, or physical exam findings. Two patients in the NAC group experienced increasing numbers of cyanotic and bradycardic episodes, which resolved when they were switched to the placebo group. One infant developed pneumothorax during NAC administration [59]. Amir et al. describe the use of nebulized 5% NAC in five premature infants with severe atelectasis. Atelectasis resolved in all patients after one to two days of treatment. No adverse effects occurred [60].

Caution is advised before drawing conclusions from most studies that have examined the efficacy of inhaled mucolytics in infants. While published case reports show sometimes dramatic improvement with the use of a medication, there is likely significant publication bias such that most unpublished cases in which the medication has been used may have demonstrated no benefit. Our anecdotal clinical experience of using dornase alfa on several occasions to treat atelectasis in the NICU has been unsuccessful, although well tolerated. Furthermore, in studies lacking a control group, it is difficult to comment with certainty if the reported clinical improvements were in fact due to the medication used. Taken together, the certainty of evidence to support use of these medications is very low, and more prospective, controlled studies are needed.

### Bronchoscopic instillation of medications

Flexible bronchoscopy may be performed to remove secretions from the airway, identify the cause of atelectasis, and potentially treat atelectasis caused by mucus plugs. In infants, an ultra-thin flexible bronchoscope is usually used, with a suction channel of only 1.2 mm in diameter. This poses a challenge for suctioning viscous secretions. To overcome this challenge, sometimes a clinician may decide to instill a mucolytic medication or normal saline directly through the working channel of the bronchoscope into the affected airway to thin secretions and facilitate their removal. Although this practice has not been formally studied in a prospective fashion in infants, several case studies and series have been reported using dornase alfa, surfactant, and NAC [61–65].

Table 1 provides a summary of studies examining the use of mucolytic medications in infants.

**Table 1:** Inhaled mucolytic medications used in infants.

Medication	Mechanism(s) of action	Evidence for efficacy	Possible adverse effects	Dose(s) used	Frequency
Dornase alfa	Hydrolyzes leukocyte-derived DNA polymers	Persistent atelectasis: - Low-quality observational/uncontrolled studies suggest efficacy [37–41] - May be more efficacious in those with at least moderate PMNs on tracheal aspirate gram stain [43] - May be more efficacious when administered with hypertonic saline [46] Prevent atelectasis: - One RCT demonstrated efficacy after cardiac surgery [44] RTIs: - Small RCT showed non statistically significant improvement in oxygenation [47]	- Large airway obstruction by mobilized secretions	Nebulized: - 1.25 mg - 2.5 mg - 1 mg/m^2^ Instilled into ETT: - 1.25 mg - 2.5 mg - <5 kg: 0.2 mg/kg - >5 kg: 1 mg/kg	- Every 8 h - Every 12 - Twice daily with 2 h between doses
3% Hypertonic Saline	- Disrupts ionic bonds within mucus gel - Dissociates DNA from mucoproteins - Increases airway surface liquid depth via osmosis - Triggers cough - Antibiofilm properties	RTIs: - One RCT demonstrated efficacy in preventing VAP, reducing duration of MV, less bacterial growth in tracheal aspirates and blood cultures [54] Persistent atelectasis: - Low quality observational data suggest efficacy [45]	- Sudden large airway obstruction by mobilized secretions - Bronchospasm - Overhydration of secretions and bronchorrhea	Nebulized: - 4 mL	- Twice daily - Every 2 h - Every 4 h - Every 6 h
7% Hypertonic Saline	Same as 3% HTS	Persistent atelectasis: - One observational study showed may be effective when used with dornase alfa [46]	- Same as 3% HTS, although risk of bronchospasm may be greater	Nebulized: - <2.5 kg: 3 mL - >2.5 kg: 4 mL	- Every 12 h
N-acetylcysteine	Cleaves disulfide bonds between mucoproteins	Maintain ETT patency: - One small RCT demonstrated no efficacy and increased airway resistance [59] - Case series suggested efficacy Persistent atelectasis: - Low quality observational data suggest efficacy [60]	- Bronchospasm - Foul odor – may cause nausea and vomiting - One pneumothorax reported	ETT instillation: - 0.1–0.2 mL - 0.25 mL of 5% NAC Nebulized: - 20 mg in 3 mL saline - 2 mL of 5% NAC	- Every hour - Every 4 h - Every 6 h - Every 8 h

*PMNs* polymorphonuclear  neutrophils, *RCT* randomized clinical trial, *RTI* respiratory tract infection, *VAP* ventilator associated pneumonia, *MV* mechanical ventilation, *HTS* hypertonic saline, *ETT* endotracheal tube.

## Inhaled antibiotics

RTIs are common in the NICU, particularly in infants requiring invasive mechanical ventilation [55]. This includes ventilator associated (bacterial) tracheitis (VAT) and VAP. While the definitions of these are somewhat controversial in neonates [55, 66–68], many research studies use the VAP criteria defined by the U.S. Center for Disease Control and Prevention (CDC). For this definition of VAP, infants must have received mechanical ventilation for at least 48 h with worsening gas exchange and at least three of the following: temperature instability, leukopenia or leukocytosis with left shift, new onset purulent sputum or change in sputum character, apnea or respiratory distress, wheezing rales or rhonchi, cough, and bradycardia or tachycardia [69]. RTIs in the NICU is associated with significant morbidity, duration of hospitalization, mortality, and healthcare costs [55, 67, 68]. Premature infants are predisposed to develop RTIs for a number of reasons, including immature immune function, recurrent respiratory tract damage and inflammation, impaired ciliary function and cough due to the presence of an ETT, bacterial colonization and biofilm formation in the ETT, and pooling of oral and gastric secretions in the oropharynx [52, 53, 55, 67, 70]. Since a wide range of organisms are implicated in causing RTIs in mechanically ventilated infants [66, 67], these patients usually receive broad spectrum systemic antibiotics, which predisposes them to adverse effects including nephrotoxicity and ototoxicity. Hence, use of antibiotics to treat RTIs may be advantageous for several reasons. Inhalation of the drug may increase antibiotic concentration delivered to the site of infection. Although not yet studied in infants, this has been demonstrated in TA of adults and in the pulmonary parenchyma in several animal studies [71–73]. Furthermore, inhalation of the antibiotic would be expected to result in less systemic exposure and hence less adverse effects [74].

The use of inhaled antibiotics in infants has only been reported in case reports and small observational studies, mostly with inhaled colistin to treat VAP. Nakwan et al. retrospectively reviewed the cases of eight neonates with VAP caused by drug-resistant *Acinetobacter baumannii* who received adjunctive nebulized colistin (with or without intravenous colistin). All patients experienced clinical cure with microbial eradication of the bacterial isolates from tracheal secretions. No clinical or laboratory adverse effects related to nebulized colistin occurred. Compared to a group of infants with VAP due to *A. baumannii* who did not receive nebulized colistin, mortality rate was decreased. However, the comparison group had a lower gestational age and birth weight, and may have been predisposed to poorer outcomes [75]. Similarly, Han Celik et al. report that inhaled colistin was effective and safe when used as an adjunct therapy to treat VAP in three neonates [76]. Choudry et al. describe the safe use of inhaled colistin (with and without intravenous colistin) in a cohort of 30 neonates with RTIs caused by multidrug-resistant organisms. They do not report on the efficacy of the treatment [77].

Hussain et al. performed a matched case-control study of infants with VAP caused by multidrug-resistant gram-negative bacteria. Sixteen patients received both intravenous and nebulized colistin, and sixteen matched patients received intravenous colistin alone. The group that received both inhaled and intravenous colistin experienced higher clinical cure rates and microbial eradication, decreased mortality, duration of intravenous colistin, and nephrotoxicity compared to the group that received intravenous colistin alone. No adverse effects of nebulized colistin such as bronchospasm were observed. A major limitation of this observational study is that the group that received intravenous colistin alone may have been more ill, as evidenced by the fact that those patients more often received multiple types of intravenous antibiotics concomitantly [78].

Inhaled colistin has also been used as monotherapy to treat VAP in preterm infants. In one retrospective study, eight preterm infants with VAP due to *A. baumannii* were treated with inhaled colistin monotherapy. All subjects experienced clinical cure and eradication of *A. baumannii* from tracheal secretions. No adverse effects were reported [79].

Doses in the above studies were either 4 mg/kg, 5 mg/kg, or 33.4 mg as a fixed dose, all given twice daily. In a pharmacokinetic study, Nakwan et al. administered a single dose of inhaled colistin (4 mg/kg) to six mechanically ventilated neonates and found that high TA concentrations (above the established minimum inhibitory concentration or MIC breakpoint) were achieved for at least twelve hours, while systemic absorption of the drug was extremely low. However, this study excluded subjects with known renal dysfunction [80]. The need for determining appropriate dosing of inhaled antibiotics for preterm infants (particularly those with renal dysfunction) is highlighted by a case report in which a patient with compromised renal function developed elevated serum tobramycin levels during treatment for pneumonia with inhaled tobramycin [81]. Currently, there is an ongoing open-label, phase 1, sequential dose escalation trial to establish preliminary tolerability, efficacy, and pharmacokinetic data for up to four different doses of inhaled tobramycin administered in the NICU. The study population consists of preterm infants with BPD who require invasive mechanical ventilation and have a pathogenic Gram-negative organism detected by TA culture (NCT04560179; *clinicaltrials.gov*).

In a pilot randomized study of infants with VAP, Levchenko et al. randomized twenty subjects to receive either IV antibiotics alone or IV antibiotics with nebulized amikacin. Duration of mechanical ventilation was shorter by one day in infants who received adjunctive nebulized amikacin. Additionally, the nebulized amikacin group experienced decreases in reactive oxygen species produced by leukocytes, leukocyte necrosis and apoptosis, and sputum microbial load [82].

## Conclusion

There is a need to evaluate the safety and efficacy of inhaled mucolytics and antibiotics in the NICU patient population. Anatomic and physiologic characteristics of infants may dramatically impact the distribution of aerosolized particles and therefore preclude extrapolation from studies of inhaled mucolytics used in older children and adults. Even within the same patient population, an inhaled drug must be assessed in spontaneously breathing infants, infants supported by invasive mechanical ventilation, and infants with different forms of underlying airway and lung disease. Multicenter collaboration is likely required to achieve adequate statistical power to perform these studies.
